# RPS19 and TYMS SNPs and Prevalent High Risk Human Papilloma Virus Infection in Nigerian Women

**DOI:** 10.1371/journal.pone.0066930

**Published:** 2013-06-27

**Authors:** Ayo Famooto, Maryam Almujtaba, Eileen Dareng, Sally Akarolo-Anthony, Celestine Ogbonna, Richard Offiong, Olayinka Olaniyan, Cosette M. Wheeler, Ayo Doumatey, Charles N. Rotimi, Adebowale Adeyemo, Clement A. Adebamowo

**Affiliations:** 1 Department of Research and Training, Institute of Human Virology, Abuja, FCT, Nigeria; 2 Department of Nutrition, Harvard School of Public Health, Boston, Massachusetts, United States of America; 3 University of Abuja Teaching Hospital, Gwagwalada, FCT, Nigeria; 4 National Hospital, Abuja, FCT, Nigeria; 5 Department of Pathology, University of New Mexico Health Sciences Center, Albuquerque, New Mexico, United States of America; 6 Center for Research on Genomics and Global Health, National Human Genome Research Institute, National Institutes of Health, Bethesda, Maryland, United States of America; 7 Department of Epidemiology and Public Health; Institute of Human Virology and Greenebaum Cancer Centre, University of Maryland School of Medicine, Baltimore, Maryland, United States of America; Sookmyung Women's University, Republic of Korea

## Abstract

High risk HPV (hrHPV) infection is a necessary cause of cervical cancer but the host genetic determinants of infection are poorly understood. We enrolled 267 women who presented to our cervical cancer screening program in Abuja, Nigeria between April 2012 and August 2012. We collected information on demographic characteristics, risk factors of cervical cancer and obtained samples of blood and cervical exfoliated cells from all participants. We used Roche Linear Array HPV Genotyping Test® to characterize the prevalent HPV according to manufacturer's instruction; Sequenom Mass Array to test 21 SNPs in genes/regions previously associated with hrHPV and regression models to examine independent factors associated with HPV infection. We considered a p<0.05 as significant because this is a replication study. There were 65 women with and 202 women without hrHPV infection. Under the allelic model, we found significant association between two SNPs, rs2305809 on *RPS19* and rs2342700 on *TYMS*, and prevalent hrHPV infection. Multivariate analysis of hrHPV risk adjusted for age, body mass index, smoking, age of menarche, age at sexual debut, lifetime total number of sexual partners and the total number of pregnancies as covariates, yielded a p-value of 0.071 and 0.010 for rs2305809 and rs2342700, respectively. Our findings in this unique population suggest that a number of genetic risk variants for hrHPV are shared with other population groups. Definitive studies with larger sample sizes and using genome wide approaches are needed to understand the genetic architecture of hrHPV risk in multiple populations.

## Introduction

With 75,000 new cases and 50,000 deaths in 2008, cervical cancer is the commonest cancer in men and women combined and among women in Sub-Saharan Africa [1]. Globally, it is the seventh most common cancer among men and women combined, and the third most common cancer in women globally [2], [3]. The disease is not uncommon in developed countries either. For example in the United States, approximately 11,000 new cases of cervical cancer were diagnosed in 2007 [4] compared with 14,500 in Nigeria which has half the population of the US [4]. Therefore while the burden of cervical cancer falls disproportionately on low resource countries; it remains a significant public health problem even in developed countries.

Persistent high risk HPV infection (hrHPV) is recognized as a necessary but not sufficient cause for Cervical Intraepithelial Neoplasm (CIN) grades 2/3 and cervical cancer (CIN2+) [5]. Several observation suggests that heritable factors have a role in HPV infection [6]. A review of 15 studies on heritability of cervical cancer risk suggested that having a first degree relative with cervical cancer increases an individual's risk by one to two fold [7]. Magnusson *et al* in a study of Swedish cancer registry data demonstrated an association between heritability and cervical cancer [8] while comparisons of cervical cancer incidence among mono- and dizygotic twins support a genetic contribution to development of cervical cancer *in situ* [9]. Another review of a potential role for genetic factors in cervical cancer *in situ* estimated the heritability to be between 11 and 15% [10], [11], [12]. Similarly the heritability of cervical carcinoma was estimated to be ∼27% (95%CI  =  26%–29%) reflecting the multifactorial nature of cervical carcinogenesis as well as the impact of environmental factors on its development [11], [12].

Improved understanding of the etiology, development of new methods of risk stratification including identification of genetic markers of hrHPV infection and CIN2+ will benefit citizens in developed and developing countries. In previous studies, SNPs in 305 genes based on a priori hypotheses of association with HPV infection and cervical cancer were evaluated and several loci of interest identified [13], [14]. Of these, genes/regions in the immune genes 2′,5′ oligoadenylate synthetase gene 3 (*OAS3*), sulfatase 1 (*SULF1*), epidermal dysplasia verruciformis (EV)-associated *EVER1* and *EVER2* genes, transmembrane channel-like 6 and 8 (*TMC6*, *TMC8*), peroxiredoxin 3 (*PRDX3*), ribosomal protein S19 (*RPS19*), interleukin 2 receptorα (*IL2RA)*, telomere maintenance 2 (*TELO2*), thymidylate synthetase (*TYMS*) and complement component 1, r subcomponent-like (*C1RL*) were associated with components of the cervical carcinogenesis pathway [14].

In this study, we sought to replicate the association between these genes/regions and risk of prevalent hrHPV infection in an African population.

## Methods

### Study participants and ethics

The study was approved by the National Health Research Ethics Committee of Nigeria.

Women were recruited from 2 cervical cancer screening clinics within the National Hospital, Abuja and University of Abuja Teaching Hospital, Abuja, Nigeria during the period April 2012 to August 2012. To be eligible to enroll in the study, the participants had to be above the age of 18 and provide written informed consent for the study. We excluded women who had had a total hysterectomy, were pregnant or could not provide an informed consent. A total number of 278 women were screened, of which 267 (96%) had complete phenotype data and were successfully genotyped (as described below). These 267 women comprised 65 women with prevalent hrHPV (cases) and 202 controls.

Using interviewer administered questionnaires, participants provided information on demographics, socio-economic status, physical activity, smoking, alcohol use, sexual and reproductive history. A gynecologic examination was carried out on each participant. During the gynecologic examination, exfoliated cervical cells were collected from the cervical os. A cervical brush was inserted into the cervical os and rotated 3 full turns to collect exfoliated cells from the cervical os. The head of the brush was subsequently snapped off and placed at the bottom of a specimen transport tube containing 95% ethanol. In addition to the gynecologic specimens collected, blood samples were also collected from the antecubital veins. All samples were transported to the IHVN laboratory. The exfoliated cervical cells were stored at −80°C until the time of analysis. Buffy coat was separated from the whole blood samples and stored till the time of analysis. Data were collected and managed using REDCap electronic data capture tools hosted at the Institute of Human Virology, Nigeria [15], [16].

### Laboratory methods

Frozen buffy coat was thawed quickly in a 37°C water bath with mild agitation and stored on ice before beginning the DNA extraction procedure. Approximately 500ul leukocyte-containing fraction was used in the extraction of germline DNA using Gentra Puregene DNA kit® according to manufacturer's instructions. Some 250 µl of DNA hydration solution was added and incubated at 65°C for 1 hour to dissolve the DNA which was then stored. DNA samples were shipped to the Center for Research on Genomics and Global Health, National Human Genome Research Institute, National Institutes of Health, Bethesda for analysis.

DNA samples were quantified using Quant-iT™ PicoGreen® dsDNA reagents and kit (Invitrogen- Life Technologies, Grand Island, NY). After quantification, DNA samples were normalized to 100 ηg/µL. To obtain the quantity of DNA required for the genotyping protocol, samples were further diluted to 3.3 ηg/µL and 2 µl of the later concentration were used to stamp the 384-well plates used for genotyping.

The SNPs were genotyped at the National Human Genome Institute (NHGRI, NIH) using iPLEX Gold assay on the MassArray platform (Sequenom, San Diego, CA) as previously described [17]. This platform allows multiplexing SNPs for moderate to high throughput genotyping and uses single-base primer extension chemistry with MALDI-TOF MS technology for detection. Briefly, the PCR and extension primers were designed using MassArray designer Software, the Sequence information were imported from public databases for all SNPs included in this study. The designed PCR and extension primers were then ordered (IDT-DNA, Coralville, IA).

The PCR reaction was at a final volume of 5 µl per well. The PCR mixture contained 10× PCR buffer, MgCl2 (25 mM), dNTPs (25 mM each), Primer mix (500 nM each) and HotStar Taq (5 U/uL). PCR conditions were as follows: denaturation at 95°C for 15 minutes, followed by 45 cycles of 20 seconds at 95°C, 30 seconds at 56°C, 1 minute at 72°C, and a final extension of 3 minutes at 72°C. Then all wells are treated with Shrimp Alkaline Phosphatase (SAP) to remove un-incorporated dNTPs. After optimization of the extension primers and adjustment of the input concentrations, the iPlex extension primer reaction was carried out to identify genotypes following the manufacturer's recommendations. Each sample was run in duplicate. A total of 29 SNPs split into 2 plexes of 13 SNPS and 16 SNPs respectively, were genotyped. The Typer 4.0 software (Sequenom) was used to process and analyze genotype calls.

The genotyping assay succeeded for 26 SNPs of the 29 SNPs tried. Four samples were excluded for having failed genotyping at 4 or more loci (i.e. sample success rate <80%). Applying further quality control filters resulted in dropping one SNP that had <90% success rate, 1 SNP for being inconsistent with Hardy-Weinberg equilibrium (HWE) expectations (P (HWE) <0.05), two SNPs for being monomorphic or invariant and one SNP with a Minor Allele Frequency (MAF) <0.05. The final association dataset comprised 21 SNPs on 267 subjects. The overall genotyping rate for this dataset was 98.7%. Concordance of duplicate genotypes was 99.9%.

HPV DNA extraction and genotyping were done from ectocervical cell samples. The HPV DNA genotyping was done using LINEAR ARRAY HPV Genotyping Test (HPV LA; Roche Diagnostics, IN) based on the principle of DNA amplification by Polymerase Chain Reaction (PCR) and nucleic acid hybridization. This genotyping test qualitatively detects 37 high- and low-risk human papillomavirus genotypes (13-high risk HPV types- 16, 18, 31, 33, 35, 39, 45, 51, 52, 56, 58, 59, 68; and 24 low-risk HPV types −6, 11, 26, 40, 42, 53, 54, 55, 61, 62, 64, 66, 67, 69, 70, 71, 72, 73, 81, 82, 83, 84, IS39, CP6108) following a four stage process of sample preparation, DNA amplification through PCR protocol using biotinylated PGMY 09/11 L1 region consensus primer, hybridization and immobilization of the amplified product to HPV genotype-related oligonucleotide probes and the capturing of detectable colored bands of amplified products on a LINEAR ARRAY HPV genotyping strip.

The ectocervical samples were into two separate Sarstead tubes. These were centrifuged at 20,000 rpm for 5 minutes to pellet the cells. The liquid supernatant was removed and the tubes were allowed to dry at either 50°C in hot oven or overnight at room temperature in a level 2 safety working cabinet. The dry cell pellets were underwent enzymatic cell lysis, denaturing by detergent and elution at high temperatures. The process isolates the human β-globin gene. The digestion solution is a mixture of 1x Proteinase K-Laureth Digestion Buffer containing proteinase K/ml, 2% (vol/vol) Laureth-12 (50 mM), Tris-HCl (pH 8.5) –1 mM EDTA. A total of 100 µL of the buffer is added to the tubes containing the dry sample. The tubes were vortexed vigorously and digested at 65°C with shaking at 1050 rpm for 30 minutes. The vortexing and digestion condition were repeated once followed by inactivation of the proteinase K lysing enzyme at 95°C for 15 minutes without shaking. Gloves were changed intermittently to avoid cross contamination of sample.

Amplification involved a pool of biotinylated primers in a PCR mastermix that specifically binds to designated sequences of nucleotide on the L1 region of the HPV genome. The primer pool which includes additional primer pair for the human β-globin allows for the amplification of HPV DNA from 37 high and low-risk human papillomavirus genotypes and β-globin gene. The amplification of the β-globin gene serves as an internal control for cervical cell adequacy, DNA extraction and amplification. A total of 5 µL of the digested DNA sample was added to 96 well PCR plate with 50 µL of HPV LINEAR ARRAY mastermix solution. The amplification was run on the conventional principle of polymerase activation and denaturing of the viral and human genomic DNA to expose the template DNA for primer to anneal to the target DNA sequence upon cooling. The polymerase extends the primers to produce complementary bases of double stranded HPV or the human β-globin DNA molecules. As the cycle continues, the target nucleic acid sequence on HPV or the human β-globin DNA is amplified. The target nucleic acid amplification in the Linear array genotyping test was enhanced by the inclusion of AmpErase enzyme and deoxyuridine triphosphate (dUTP) in the master mix. The addition of denaturing solution and high temperature above 55°C during the amplification inactivates the AmpErase and prevents the degradation of the target amplicon by the enzyme. The amplification was performed on Applied Biosystems Gold-plated 96-well GeneAmp PCR System 9700 as specified by the manufacturer. Appropriate volumes for controls of contamination and assay sensitivity were added in the 96-well assay.

The hybridization assay was performed on Tecan ProfiBlot-48 robots (Tecan, Austria) using the Roche HPV Linear Array detection solutions. The samples were the denatured HPV or the human β-globin amplification products. Each Linear Array HPV genotyping strip coated with HPV, human β-globin and cross-reactive oligonucleotide probes (for HPV genotype 33, 35, 52 and 58) was placed on wells of the hybridization tray. Denatured biotin labelled amplicons and hybridization buffer were also transferred to the wells. As the reaction progresses with continuous but slow agitation rate, the amplicons sequence hybridizes and matches complementary oligonucleotide probes on the strip.

The strips were automatically washed to remove any unbound material followed by binding of Streptavidin-Horseradish Peroxidase conjugate (SA-HRP) to the strips. A second wash removes unbound SA-HRP. The SA-HRPcatalyzes 3,3′,5,5′ -tetramethylbenzidine (TMB) in the presence of Hydrogen peroxide to form detectable blue colour complex that emerges at the point of hybridization. Each HPV type was detected visually by a type-specific probe masked on the genotyping strip except for HPV 52. The detection of the latter depends on a probe that cross hybridizes with HPV 33, 35, 52 and 58. HPV 52 can only be inferred if the cross-reactive probe is hybridized in the absence of any of the HPV 33, 35 and 58 type-specific probes. The HPV genotypes strip result was interpreted by two independent readers across a reference template supplied by the manufacturer along with the detection kit. The consensus result was compared by a third reviewer with custom computer application result reader.

### Statistical analysis

Association analysis was done using PLINK version 1.07 [18]. Two models were considered. In the first model, association was done under an allelic model. This model is a case-control analysis that compares allele frequencies between cases and controls with no adjustment for covariates. The second model was a logistic model adjusting for age, body mass index (BMI), smoking, age at menarche, age at sexual debut, lifetime total number of sexual partners and total number of pregnancies had, assuming an additive genetic model. Given the prior information about the role of the tested variants in hrHPV, we considered this study a replication study, therefore, nominal *p*-values <0.05 were considered significant.

## Results

**Figure 1:**
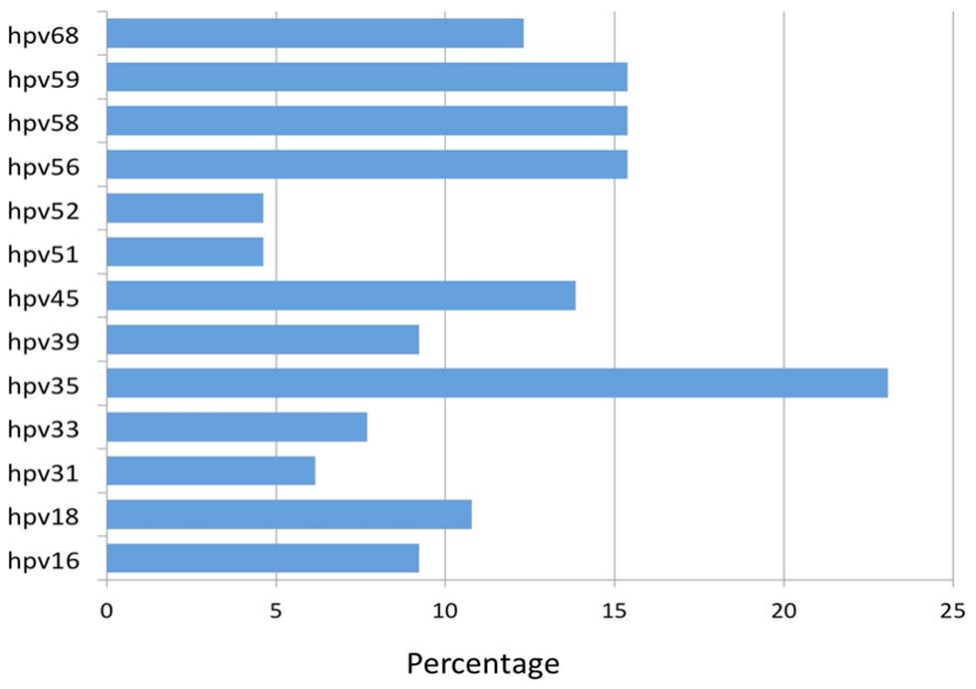
Distribution of hrHPV types among Nigerian women.

There were 267 women, 65 with hrHPV and 202 controls enrolled in this study. The characteristics of the participants are shown in Table 1. Participants with hrHPV were slightly younger and had lower BMI than controls (Table 1). Cigarette smoking was uncommon in this population, with only three percent of participants reporting smoking. The distribution of the hrHPV types is shown in Figure 1. The most common hrHPV types were HPV35 found in 15 women (23.1%), HPV59, HPV58 and HPV56 each found in 10 women (15.4%), HPV45 found in 9 women (13.9%) and HPV68 found in 8 women (12.3%). The others were HPV18 in 7 women (10.8%), HPV39 in 6 (9.2%), HPV16 in 6 (9.2%), HPV33 in 5 (7.7%), HPV31 in 4 (6.2%), and HPV52 and HPV51, each in 3 women (4.6%). Forty-four (67.7%) of the 65 hrHPV positive subjects had a single hrHPV type. The other 21 (32.3%) had two or more hrHPV types, comprising 15 subjects with two types, four with three types and one subject each with 4 types and 6 types, respectively.

**Table 1:** Characteristics of the study participants.

Characteristic	Cases	Controls	*P value* *
N	65	202	
Age (years), Mean ± SD	34.3± (7.4)	37.6 (7.7)	0.002
BMI (kg/m^2^), Mean ± SD	25.0± (5.1)	27.6 (6.0)	0.0008
Smoking (%)	1.5	3.5	0.425
Age at menarche (years)#	15 (14, 16)	15 (13, 15.5)	0.015
Age at sexual debut (years) #	19 (16, 23)	19 (18, 22)	0.606
Number of pregnancies had#	2 (0, 3.5)	2 (1, 3)	0.595
Total number of sexual partners (lifetime) #	3 (1,4)	3 (1,4)	0.723

# Median (25^th^ percentile, 75^th^ percentile).

* *P* value for Student t (age, BMI), chi-square (smoking) or Wilcoxon rank sum (all other variables) tests.

Two SNPs, rs2305809 (*RPS19*) and rs2342700 (*TYMS*), were significant under the allelic model (Table 2). Adjusting for age, BMI and smoking made the association stronger for rs2342700, with the p-value going from 0.037 to 8.91×10^−3^ (data not shown). Further adjustment of the model for the age of menarche, age at sexual debut, lifetime total number of sexual partners and the total number of pregnancies yielded a p value of 0.071 and 0.010 for rs2305809 and rs2342700, respectively (Table 2). Notably, both of these SNPs are highly differentiated between African ancestry and European ancestry populations (with frequencies rs2305809 (T) 0.045 in YRI *versus* 0.505 in CEU, rs2342700(C) 0.258 YRI *versus* 0.642 CEU). This suggests that these two SNPS are significantly associated with hrHPV across multiple ethnic groups despite allele frequency differences. The association findings for all 21 SNPs tested are presented in Table 2.

**Table 2:** SNPs tested for association with hrHPV infection among Nigerian women.

Rank	SNP	Gene [ref]	Chromosome	Minor Allele	MAF Cases	MAF Controls	Allelic Model		Logistic Model	
							OR and 95% CI	P-value	OR and 95% CI	P-value
1	rs2305809	RPS19 [18]	19q13.2	T	0.0385	0.0990	0.3640 (0.1405, 0.9427)	0.0306	0.3936 (0.1428, 1.0850)	0.0714
2	rs2342700	TYMS [18]	18p11.32	C	0.3154	0.4183	0.6406 (0.4211, 0.9744)	0.0366	0.5328 (0.3293, 0.8619)	0.0103
3	rs4786772	TELO2 [18]	16p13.3	G	0.3387	0.4313	0.6753 (0.4411, 1.0340)	0.0699	0.7410 (0.4666, 1.1770)	0.2040
4	rs6926723	GTF2H4 [17]	6p21.3	A	0.0806	0.1218	0.6323 (0.3098, 1.2910)	0.2047	0.5523 (0.2415, 1.2630)	0.1596
5	rs5757133	DMC1 [17]	17q25.1	T	0.1077	0.0767	1.4520 (0.7471, 2.8230)	0.2690	1.6050 (0.7674, 3.3570)	0.2089
6	rs11177074	IFNG [17]	12q14	C	0.2231	0.1856	1.2600 (0.7770, 2.0420)	0.3485	1.0830 (0.6119, 1.9170)	0.7843
7	rs4737999	SULF1 [17]	8q13.3	A	0.0937	0.0693	1.3890 (0.6846, 2.8190)	0.3608	1.1170 (0.5129, 2.4320)	0.7809
8	rs17132382	POLN [17]	4p16.2	T	0.4462	0.4901	0.8381 (0.5635, 1.2470)	0.3830	0.7656 (0.4946, 1.1850)	0.2306
9	rs12307655	OAS1 [17]	12q24.2	C	0.3846	0.4254	0.8443 (0.5632, 1.2660)	0.4124	0.8858 (0.5534, 1.4180)	0.6136
10	rs7251	IRF3 [18]	19q13.3-q13.4	C	0.2891	0.2562	1.1800 (0.7579, 1.8380)	0.4629	1.1300 (0.6901, 1.8520)	0.6264
11	rs10108002	SULF1 [17]	8q13.3	T	0.1231	0.1015	1.2430 (0.6719, 2.2980)	0.4880	1.0350 (0.5015, 2.1370)	0.9253
12	rs2239359	FANCA [18]	16q24.3	C	0.2422	0.2662	0.8811 (0.5557, 1.3970)	0.5903	0.8230 (0.4994, 1.3560)	0.4446
13	rs16970849	TMC8 [17]	17q25	A	0.2656	0.2438	1.1220 (0.7129, 1.7660)	0.6187	1.0900 (0.6514, 1.8250)	0.7423
14	rs2290907	TNRC6C [17]	17	C	0.4000	0.4229	0.9098 (0.6081, 1.3610)	0.6455	1.0410 (0.6518, 1.6640)	0.8651
15	rs7190823	FANCA [18]	16q24.3	T	0.2462	0.2277	1.1070 (0.6978, 1.7570)	0.6651	1.1040 (0.6533, 1.8650)	0.7121
16	rs2894054	GTF2H4 [17]	6p21.3	A	0.1769	0.1683	1.0620 (0.6312, 1.7870)	0.8204	1.2110 (0.6733, 2.1780)	0.5228
17	rs12302655	OAS3 [17]	12q24.2	A	0.4692	0.4802	0.9570 (0.6441, 1.4220)	0.8276	1.1570 (0.7320, 1.8280)	Q
18	rs3784621	DUT [17]	15q15-q21.1	T	0.3769	0.3663	1.0460 (0.6955, 1.5740)	0.8278	0.9314 (0.6013, 1.4430)	0.7502
19	rs2476491	IL2RA [18]	10p15-p14	T	0.1102	0.1173	0.9313 (0.4846, 1.7900)	0.8308	1.0070 (0.5138, 1.9730)	0.9840
20	rs718802	OAS2 [17]	12q24.2	C	0.4683	0.4722	0.9842 (0.6586, 1.4710)	0.9380	0.9579 (0.6069, 1.5120)	0.8535
21	rs7195066	FANCA [18]	16q24.3	C	0.3047	0.3021	1.0120 (0.6554, 1.5640)	0.9557	0.8788 (0.5415, 1.4260)	0.6009

CI  =  Confidence Interval.

## Discussion

In this study of genetic risk of prevalent hrHPV infections in Nigerian women, we found significant associations with SNPs on ribosomal protein gene S19 (*RPS19*) and Thymidylate Synthase gene (*TYMS*), in the allelic model. This risk remained significant, after adjusting for age, body mass index, smoking, age at menarche, age at sexual debut, lifetime total number of sexual partners and the total number of pregnancies.

*RPS19* is a ribosomal protein expressed by hematopoietic and non-hematopoietic tissues. Mutations of this ribosomal gene have been associated with Diamond-Blackfan anemia. Individuals with this form of anemia have been shown to have increased risk of malignancies including osteosarcoma, breast, liver, gastric cancers and hematological malignancies (acute myeloid and lymphoblastic leukemia, lymphoma, myelodysplasia) [19], [20], [21], [22]. In one previous study, *RPS19* has also been shown to be associated with the risk of cervical cancer and persistence of HPV [14]. Our results confirm this previous finding that a SNP on RPS19 is associated with risk of hrHPV infection. The mechanism of this association is not yet known and requires further study.

TYMS, the protein product of the *TYMS* gene, catalyzes the synthesis of thymidylate, or deoxythymidine monophosphate (dTMP), from deoxyuridine monophosphate (dUMP) with 5,10-methylenetetrahydrofolate as the methyl donor [23]. dTMP is subsequently phosphorylated to thymidine triphosphate, which is used for DNA synthesis and repair. Enhanced TYMS activity has been shown to be associated with reduced risk for hepatocellular cancer [23]. We found an association between a SNP in this gene and risk of hrHPV infection. This finding confirms the report by Safaeian *et al* and together with the *RPS19* finding raises innovative questions about biological pathways that may be associated with hrHPV and possibly other viral infections [13], [14].

This is the first study investigating genetic determinants of hrHPV infections in Africans and while we confirm 2 of the previously reported genetic risk factors, we did not find any association with the other SNPs that had been previously reported. Our results supports a hitherto unsuspected role for ribosomal and mitochondrial processes in hrHPV infection. It is possible that such processes play a role in other viral oncogenes or are uniquely related to hrHPV infection.

Our study has several limitations. We evaluated association between these SNPs and prevalent hrHPV infection which has a weaker association with cervical cancer and precancer. However, we continue to follow up these participants and will be able to evaluate whether these associations change with persistence of hrHPV infection in future. We were not able to evaluate the association of *RPS19* and *TYMS* with specific types of hrHPV because of small sample size. Since our study is continuing, we should be able to do this in future. We focused on hrHPV infection in this paper and did not consider cervical cancer or precancer. While these genetic factors may be associated with hrHPV infection, they may not be or have very weak association with cervical cancer. Nevertheless, given the strong association between hrHPV infection and cervical carcinogenesis, they may contribute to understanding the biological mechanisms and risk stratification.

In conclusion, we present novel findings of the genetic risk factors for hrHPV infection among African women. Although several risk factors for hrHPV infection have been established, there has been little work on the genetic risks. Our findings support the use of hypothesis free methods to evaluate these risk factors particular in the African population which is characterized by marked genetic heterogeneity.
